# 

**Published:** 1916-04-01

**Authors:** 


### Miss A. J. Weighall, R.R.C.

AMONGST the sisters trained at the Bristol Royal Infirmary who are doing war work, whose names we gave in our article in last week's issue, "Patriotism and the Nurse-Training Schools," we omitted to mention the name of Miss A. J. Weighall, R.R.C., who joined the Q.A.I.M.N.S. Reserve at the beginning of the war. Sister Weighall was called up for duty in the Indian General Hospital, Royal Pavilion, Brighton, and afterwards at the Royal Herbert Hospital, Woolwich. She has since been appointed Lecturer in Red Cross Nursing, and is also doing other war work.

### Annua Meetings of Special Interest.

HOSPITAL SATURDAY FUND at the Mansion House, Wednesday, April 12, 1916, at 7.30 P.M. The Right Hon. the Lord Mayor in the chair.

**An Address will be given** by Sir Alfred Pearce Gould on "The Effects of the War on Work in Civil Hospitals."

### PASSING EVENTS AND TOPICS.

### Motor Boats for the Tigris.

THREE hospital motor launches have been presented to the British Red Cross Society by the Buckinghamshire branch of the Society. They were recently named by Princess Alexander of Teck at Romney Lock, Windsor.

### The Hospital Ship "Sunbeam."

AT Bombay, on March 20, Earl Brassey publicly presented his famous yacht *Sunbeam* to Captain Landseer, Director of the Royal Indian Marine, representing the Government of India, for use as a hospital ship.

### Royal Orthopædic Hospital.

LAST year 1,162 wounded soldiers passed through this hospital. Presiding at the annual meeting of governors, Mr. J. R. Cooper said that New Zealand, Queensland, and Trinidad had sent nearly £5,000 worth of food.

### A Nurse Cavell Memorial.

ON March 21 the Lord Mayor presented to the acting chairman of the London Hospital, Mr. W. Douro Hoare, a cheque for £10,031, which had been raised by the *Daily Mirror* and its readers as a memorial to Nurse Cavell.

### The Italian Hospital.

THE annual meeting of the Italian Hospital in London was held on March 22, Prince Livio Borghese presiding. Sir Samuel Coats, chairman of the board of management, said that about sixty beds had been added for the use of wounded Belgian and British soldiers.

### Motor Ambulances for France.

THE British Ambulance Committee is making an appeal for motor ambulances to replace those worn out by work on the French front. Donations may be forwarded to the president, the Duke of Portland, British Ambulance Committee, 23A Bruton Street, London, W.

### A Hospital Scheme Abandoned.

THE scheme for the erection of a hospital with accommodation for 2,000 wounded soldiers in Platt Fields Park, Manchester, has been dropped, the military authorities having arrived at the conclusion that the expenditure necessary to adapt the site to its purpose is too large.

### Gift of a Mansion and Grounds.

THE Manchester Children's Hospital has received a splendid gift from Mr. Samuel Bramwell. Taddington Hall, a Peakland mansion, together with its grounds, has been placed at the disposal of the governors for conversion into a convalescent home for Manchester children, or to be used in any other way, or disposed of as the governors wish.

### Samaritan Hospital for Women, Liverpool.

AT the annual meeting of the Samaritan Hospital for Women, Upper Parliament Street, Liverpool, held on March 22, it was stated that there had been an increase of over £100 in contributions from patients. The committee attribute this to the higher earnings of the class of citizens from which the patients are drawn.

### A Will before Operation.

THE house committee of the Royal Infirmary, Bradford, had an encouraging, though rather unusual, circumstance reported to them last week. A patient who had been admitted to undergo a serious surgical operation made his will after entering the hospital and bequeathed the sum of £50 to it. The patient died a few days after the operation was performed, and his executor has informed the infirmary authorities that the deceased had no near relatives, and the estate will not realise a large amount.

### Poplar Hospital.

AT the annual meeting of the governors on March 21, a vote of sympathy was passed with Lady Knutsford on the serious accident to Lord Knutsford, who is chairman of the hospital. The annual report which was presented at the meeting stated that the income for the year was £19,858, by far the largest ever received, except in 1910, when the amount of £20,683 included a legacy of £6,000. In addition to the wounded soldiers admitted as in-patients, 480 were treated in the casualty and out-patients' departments last year.

### HOSPITAL RESULTS IN 1915.

### More Early Reports of the Voluntary Hospitals.

MOUNT VERNON HOSPITAL.—A word of congratulation is due to Mr. W. J. Morton in respect of the attractive and convenient form taken by the latest report. It weighs two and a half ounces. So far as we can discover, no important detail of the work of the hospital (with the exception of the medical returns, which are no doubt issued separately) has been omitted. Nothing essential has been lost by economy in paper, printing, and postage. The ordinary income falls short of the expenditure by about £800, and what legacies were received were used for current expenses. The loss of a number of subscribers is announced. There are several attractive photographs in the report.

LEICESTER ROYAL INFIRMARY.—The report weighs thirteen ounces, is printed on stout paper, the subscribers' names and addresses are in large type, and run to about fifteen lines to the page. There are 266 pages of matter in the issue which, as hospital reports go, must be quite an expensive publication. The ordinary income, £28,368, fell short of the expenditure by about £300. The cost of provisions in 1915 was £2,000 more than in 1914. Under "Surgery and Dispensary" the cost was about equal. The number of out-patients fell from 35,091 in 1914 to 14,910 in 1915.

GREAT NORTHERN CENTRAL HOSPITAL.—Fifty doctors, nurses, clerks, and porters are now serving in the Naval and Military Forces. In addition to 626 soldiers received in the wards, 12,000 men training in this country have been treated in the out-patient and casualty departments. The total income amounted to £24,748, and the expenditure was £24,305 (as compared with £20,789 in 1914). The hospital is unfortunate that its diamond jubilee falls in this unsettled year. Such an occasion could in ordinary times be made much use of to place the institution on a sound financial basis and to further the wishes of the governors that an adequate nurses' home shall be provided.

Rates of Subscription: Twelve months, 6/6; Six months, 4/-; Three months, 2/-, post free to any address in the United Kingdom. Abroad, Twelve months, 8/8; Six months, 5/-; Three months, 2/6, post free.—Address The Subscription Dept., "The Hospital," The Hospital Building, 28/29 Southampton Street, Strand, London, W.C.

